# Modulation of the effects of class Ib antiarrhythmics on cardiac Na_V_1.5-encoded channels by accessory Na_V_**β** subunits

**DOI:** 10.1172/jci.insight.143092

**Published:** 2021-08-09

**Authors:** Wandi Zhu, Wei Wang, Paweorn Angsutararux, Rebecca L. Mellor, Lori L. Isom, Jeanne M. Nerbonne, Jonathan R. Silva

**Affiliations:** 1 Department of Biomedical Engineering, McKelvey School of Engineering, Washington University in St. Louis, St. Louis, Missouri, USA.; 2 Department of Medicine, Brigham and Women’s Hospital, Boston, Massachusetts, USA.; 3 Department of Internal Medicine, Washington University School of Medicine in St. Louis, St. Louis, Missouri, USA.; 4 Department of Pharmacology, University of Michigan Medical School, Ann Arbor, Michigan, USA.; 5 Department of Developmental Biology, Washington University School of Medicine in St. Louis, St. Louis, Missouri, USA.

**Keywords:** Cardiology, Therapeutics, Arrhythmias, Drug therapy, Sodium channels

## Abstract

Native myocardial voltage-gated sodium (Na_V_) channels function in macromolecular complexes comprising a pore-forming (α) subunit and multiple accessory proteins. Here, we investigated the impact of accessory Na_V_β1 and Na_V_β3 subunits on the functional effects of 2 well-known class Ib antiarrhythmics, lidocaine and ranolazine, on the predominant Na_V_ channel α subunit, Na_V_1.5, expressed in the mammalian heart. We showed that both drugs stabilized the activated conformation of the voltage sensor of domain-III (DIII-VSD) in Na_V_1.5. In the presence of Na_V_β1, the effect of lidocaine on the DIII-VSD was enhanced, whereas the effect of ranolazine was abolished. Mutating the main class Ib drug-binding site, F1760, affected but did not abolish the modulation of drug block by Na_V_β1/β3. Recordings from adult mouse ventricular myocytes demonstrated that loss of *Scn1b* (Na_V_β1) differentially affected the potencies of lidocaine and ranolazine. In vivo experiments revealed distinct ECG responses to i.p. injection of ranolazine or lidocaine in WT and *Scn1b*-null animals, suggesting that Na_V_β1 modulated drug responses at the whole-heart level. In the human heart, we found that *SCN1B* transcript expression was 3 times higher in the atria than ventricles, differences that could, in combination with inherited or acquired cardiovascular disease, dramatically affect patient response to class Ib antiarrhythmic therapies.

## Graphical abstract

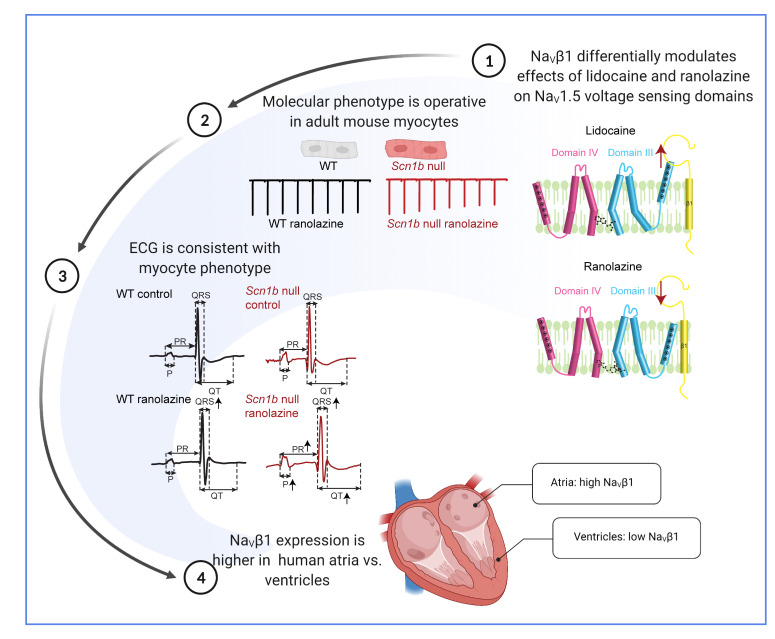

## Introduction

Inward Na^+^ currents (I_Na–_) carried by voltage-gated (Na_V_) channels underlie the initiation and propagation of action potentials in the atria and ventricles (1). Functional Na_V_ channels reflect the assembly of the 4 homologous domains (DI–DIV) in the pore-forming (α) subunit that are connected by intracellular linkers. Each domain contains 6 transmembrane segments (S1–S6). S1–S4 form the voltage-sensing domains (VSDs). The VSDs undergo conformational changes upon membrane depolarization, which open the pore (S5–S6), enabling inward Na^+^ flux (2). Native myocardial Na_V_ channels function in macromolecular protein complexes, containing many regulatory and anchoring proteins that differentially affect channel function and localization based on the cell type (3). Na_V_β subunits are essential components of these macromolecular complexes. There are 5 different types of Na_V_β subunits, Na_V_β1, Na_V_β1B, Na_V_β2, Na_V_β3, and Na_V_β4. Na_V_β1, Na_V_β1B, and Na_V_β3 interact with the Na_V_ α subunits noncovalently; Na_V_β2 and Na_V_β4 are linked covalently through the formation of disulfide bonds (4). Na_V_β1, Na_V_β2, Na_V_β3, and Na_V_β4 are transmembrane proteins, whereas Na_V_β1B is secreted (5). Consistent with a crucial role for Na_V_β subunits in maintaining normal heart function, variants in the genes encoding Na_V_β subunits have been linked to cardiac rhythm disorders, including Brugada syndrome, long QT syndrome, and sick sinus syndrome (4). However, recent evidence suggests that SCN1B may not be a monogenic cause of Brugada or sudden arrhythmic death syndrome (6, 7). Na_V_β1 and Na_V_β1B, splice variants of *SCN1B*, are the dominant Na_V_β subunits in the mammalian heart (8).

Although Na_V_β subunits were first cloned from a rat brain in the 1990s (9), the molecular interactions between Na_V_α-Na_V_β subunits have remained elusive until recently. The cryo-electron microscopy structures of the Na_V_1.4-Na_V_β1 and the Na_V_1.7-Na_V_β1-Na_V_β2 complexes suggest that Na_V_β1 coassembles with Na_V_ α subunits near the domain-III VSD (DIII-VSD) (10–12). However, the recent structure of Na_V_1.5 revealed that Na_V_β1 interacts with the predominant cardiac Na_V_ α subunit at a distinct site or sites that are characterized by weaker binding and an unresolvable Na_V_1.5-Na_V_β1 complex (13). This difference in comparison with channels encoded by other Na_V_ α subunits is partially due to the unique N-linked glycosylation of Na_V_1.5 that hinders its interaction with the Ig domain of Na_V_β1 (13). Intriguingly, Na_V_β1 and Na_V_β3 are highly homologous except in the Ig domains. Previously, optical tracking of the Na_V_1.5 VSDs using voltage-clamp fluorometry (VCF) revealed that the Na_V_β3 subunit modulates both the DIII and the DIV-VSDs, whereas Na_V_β1 only modulates the DIV-VSD conformational dynamics (14, 15). Fluorescence quenching experiments showed that the DIII-VSD is in close proximity to Na_V_β3 but not Na_V_β1 (14). These results suggest that Na_V_β1 and Na_V_β3 regulate the Na_V_1.5 DIII-VSD differently.

The conformational changes in the VSDs are not only important for regulating channel gating; they are also essential for modulating channel interactions with drugs, including those that bind to the pore domain, such as local anesthetics (16). Previously, VCF and gating current recordings showed that when lidocaine blocks Na_V_1.4 channels, it stabilizes the DIII-VSD in its activated conformation (17). Moreover, we recently demonstrated that alteration of DIII-VSD conformational changes caused by long QT syndrome 3 variants leads to channels with different mexiletine sensitivities (18, 19).

Class I antiarrhythmics modulate cardiomyocyte excitability via Na_V_ channel targeting. Class Ib molecules, such as lidocaine, ranolazine, and mexiletine, specifically modulate the late component of I_Na_, resulting in shortening of the action potential duration in ventricular cardiomyocytes (20). Lidocaine has long been used to manage ventricular arrhythmias in hospital settings (21). Ranolazine has been shown to be effective in controlling various cases of atrial fibrillation (AF) (22–24), particularly paroxysmal AF (25, 26). Recently, the RAID trial demonstrated that ranolazine also marginally lowered the risk of recurrent ventricular tachycardia and ventricular fibrillation in high-risk patients with implanted cardioverter-defibrillators (27). Although both drugs are commonly prescribed for several arrhythmias, their efficacies are highly variable. Thus, it remains an important task to understand the determinants of channel-drug interactions that contribute to this variability.

In the experiments presented here, we aimed to understand the molecular mechanisms whereby noncovalently bound Na_V_β subunits modulate the interaction of class Ib antiarrhythmics with myocardial Na_V_1.5 channels. We further investigated the physiological significance of this modulation by assessing ranolazine and lidocaine drug blockade of native Na_V_ currents in mouse ventricular myocytes, probing the mRNA expression levels of Na_V_β subunits in human hearts and detailing the in vivo electrophysiological phenotypes evident in the cardiac-specific *Scn1b*-null mouse (28). Our results showed a critical role for β subunits in differentially modulating the efficacy of lidocaine and ranolazine, implying that patient-to-patient differences in β subunit expression are likely to have a significant impact on therapeutic outcomes.

## Results

### Both lidocaine and ranolazine alter Na_V_1.5 DIII-VSD dynamics.

Previous studies demonstrated that lidocaine shifts the activation of the DIII-VSD in rat Na_V_1.4 channels encoded by *Scn4a* and prominent in skeletal muscle in the hyperpolarizing direction (16, 29, 30). Recent findings showed that a class Ib antiarrhythmic, mexiletine, which is similar in structure to lidocaine, also affects the DIII-VSD conformation in Na_V_1.5 channels (18, 19). The DIII-VSD effect also determines the tonic and use-dependent properties of class Ib drugs (18, 19). Taken together, these observations suggest that factors that alter drug effects on the DIII-VSD would be expected to have an impact on therapeutic efficacy.

**Figure 1:**
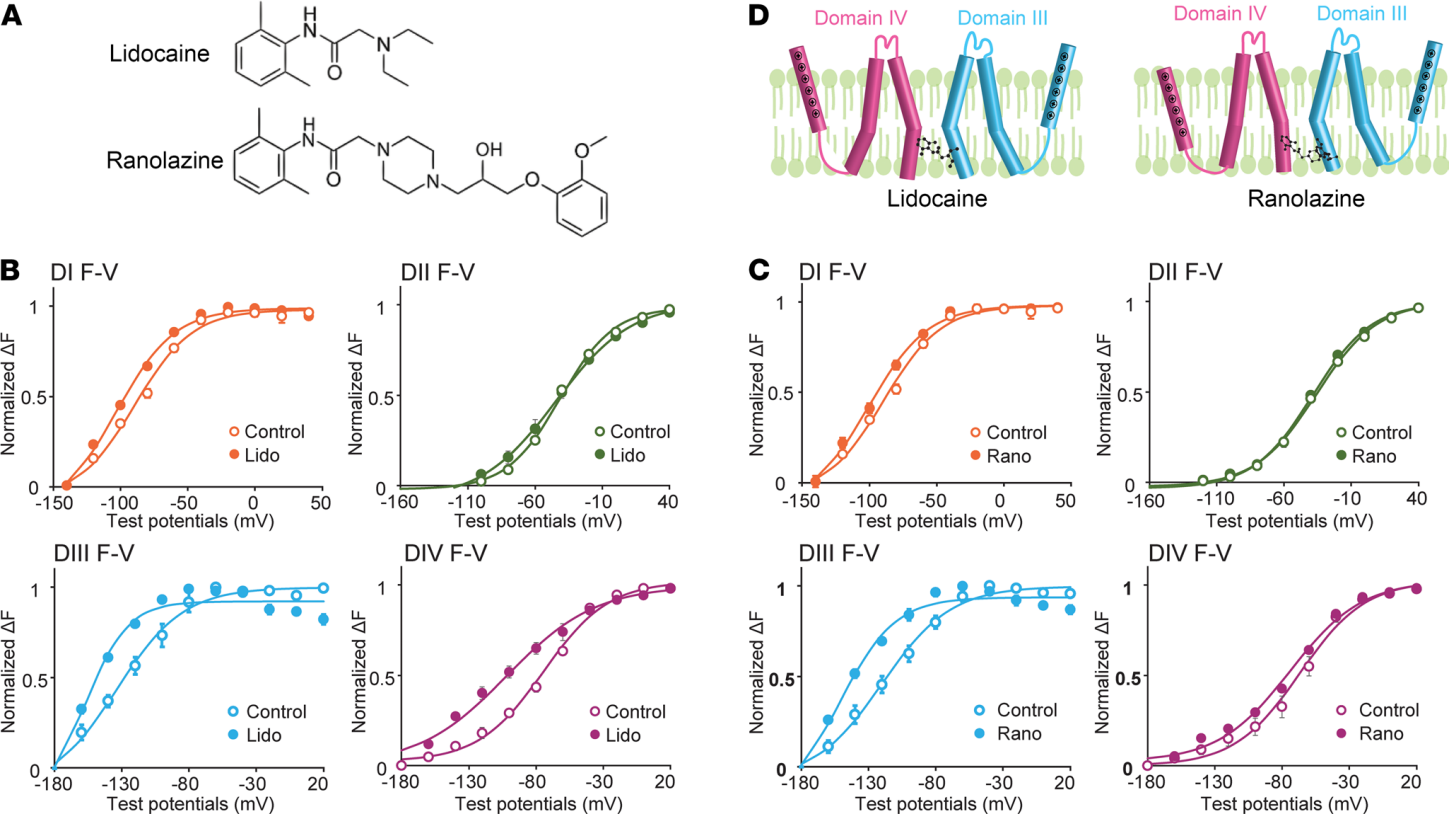
Class Ib antiarrhythmics lidocaine and ranolazine alter Na_V_1.5 VSD conformations. (**A**) Chemical structures of lidocaine and ranolazine. (**B**) Voltage dependence of the steady-state fluorescence (F-V curves) from the 4 domains (DI-V215C, DII-S805C, DIII-M1296C, DIV-S1618C) of Na_V_1.5 before and after 10 mM lidocaine. Lidocaine was used at 10 mM to produce a robust tonic block (TB). In the presence of lidocaine, fluorescence was measured when TB reached more than 70%. Lidocaine induced a hyperpolarizing shift in both the DIII and DIV F-V curves. (**C**) F-V curves from the 4 domains of Na_V_1.5 before and after 4 mM ranolazine. Similar to the lidocaine experiment (**B**), in the presence of ranolazine, the fluorescence was measured when TB reached more than 70%. Ranolazine caused a hyperpolarizing shift in the DIII but not in the DIV F-V curve. (**D**) Schematic showing effects of lidocaine and ranolazine on the DIII- and DIV-VSDs; note that each VSD is represented by a single S4 segment for clarity. Lidocaine caused both the DIII and the DIV VSDs to stabilize in the activated conformation, whereas ranolazine only stabilized the DIII-VSD in the activated position. Each data set represents mean ± SEM values from 4–6 cells.

To explore this hypothesis, we first used VCF to assess the effects of 2 class Ib antiarrhythmics, lidocaine and ranolazine (Figure 1A), on the DIII-VSD in heterologously expressed human Na_V_1.5 channels, which are encoded by *SCN5A*, the predominant Na_V_ α subunit expressed in the mammalian heart (Figure 1, B and C). When we expressed the Na_V_1.5 α subunit alone in *Xenopus* oocytes, we observed a hyperpolarizing shift (ΔV_1/2_ = –24.8 ± 9.4 mV, *P* = 0.03) in the DIII fluorescence-voltage (F-V) curve on exposure to 10 mM lidocaine, and a similar shift (ΔV_1/2_ = –30.7 ± 7.5 mV, *P* = 0.05) on application of 4 mM ranolazine, suggesting that both lidocaine and ranolazine stabilize the DIII-VSD in its activated conformation (Figure 1, B–D). The observation of similar effects on the DIII-VSD caused by both drugs is not surprising because they share similar molecular structures (Figure 1A), shown previously to interact with residue F1760 in DIV-S6 (20, 31). In addition, however, the effects of lidocaine and ranolazine are not identical. Lidocaine, for example, induced a hyperpolarizing shift in the DIV F-V curve, an effect not observed with ranolazine (Figure 1, B–D), suggesting that despite sharing common binding motifs on the Na_V_1.5 α subunit, the distinct chemical structures of lidocaine and ranolazine (Figure 1A) uniquely regulate DIV-VSD dynamics.

### Na_V_β1 and Na_V_β3 differentially modulate lidocaine/ranolazine effects on the DIII-VSD.

We have previously shown that both Na_V_β1 and Na_V_β3 alter DIII-VSD dynamics during Na_V_1.5 channel gating (14). Thus, we hypothesized that these Na_V_β subunits will also alter the effects of class Ib antiarrhythmics on the DIII-VSD. To test this hypothesis, we coexpressed Na_V_1.5 with the Na_V_β1 or Na_V_β3 subunit and measured DIII-VSD and DIV-VSD conformational changes before and after lidocaine or ranolazine application.

**Figure 2:**
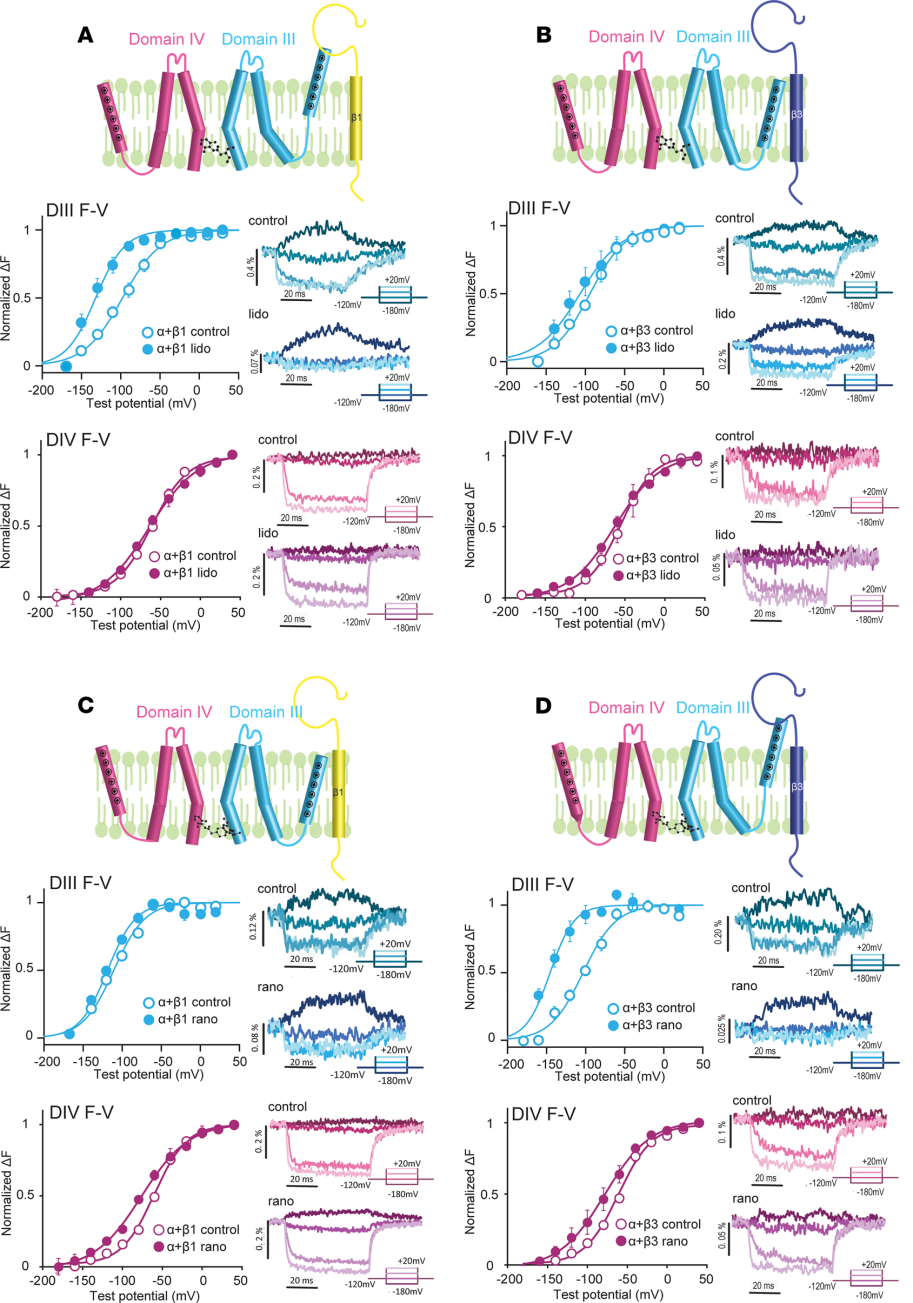
Coexpression with Na_V_β1 or Na_V_β3 differentially modulates the effect of lidocaine and ranolazine on the DIII-VSD. (**A**) In the presence of Na_V_β1, the hyperpolarizing shift in the DIII F-V curve produced by lidocaine was enhanced compared with the Na_v_1.5 α subunit expressed alone. In marked contrast, the DIV F-V curve was not affected by lidocaine with Na_V_β1 present. (**B**) In contrast with Na_V_β1 (**A**), the hyperpolarized shift in the DIII F-V curve induced by lidocaine was eliminated when Na_V_β3 was coexpressed. Similar to Na_V_β1, however, the DIV F-V curve was minimally affected by lidocaine. (**C**) In the presence of Na_V_β1, the effect of ranolazine on the DIII F-V curve was eliminated, whereas the DIV F-V was slightly hyperpolarized. (**D**) In contrast with Na_V_β1 (**C**), the hyperpolarized shift in the DIII F-V curve caused by ranolazine was enhanced when Na_V_β3 was coexpressed. In the presence of Na_V_β3, ranolazine also caused a small hyperpolarizing shift in the DIV F-V curve. Each data set represents mean ± SEM values from 4–6 cells.

When we coexpressed Na_V_1.5 with Na_V_β1, we observed distinct DIII-VSD responses to lidocaine and ranolazine. Lidocaine induced a greater hyperpolarizing shift (ΔV_1/2_ = –57.6 ± 10.2 mV, *P* = 0.01) in DIII-FV (Figure 2A) when Na_V_β1 was present compared with the Na_V_1.5 α subunit expressed alone. Exposure to ranolazine, in marked contrast, did not result in a significant DIII-FV shift (ΔV_1/2_ = –12.8 ± 16.8 mV, *P* = 0.53) (Figure 2C), suggesting that the DIII-VSD was free to move in Na_V_1.5 channels in the presence of Na_V_β1 to recover to the resting state. Although the presence of Na_V_β1 increased the lidocaine effect on the DIII-VSD, Na_V_β1 coexpression eliminated the ranolazine effect.

Strikingly, coexpression with the Na_V_β3 subunit resulted in opposite effects on lidocaine and ranolazine interaction with the DIII-VSD. Upon lidocaine block, the DIII F-V curve was minimally shifted to more hyperpolarized potentials (ΔV_1/2_ = –25.3 ± 10.9 mV, *P* = 0.13) (Figure 2B), while the ranolazine effect on the DIII-VSD was potentiated, resulting in a larger hyperpolarizing shift in the DIII F-V (ΔV_1/2_ = 58.0 ± 4.7 mV, *P* < 0.001) (Figure 2D).

Additionally, coexpression of Na_V_β1 or Na_V_β3 with Na_V_1.5 both eliminated the hyperpolarizing shift in the DIV F-V curve that was observed with the Na_V_1.5 α subunit expressed alone (Figure 2, A and B), suggesting that the Na_V_β1 and Na_V_β3 subunits similarly altered lidocaine’s effect on the DIV-VSD.

**Figure 3:**
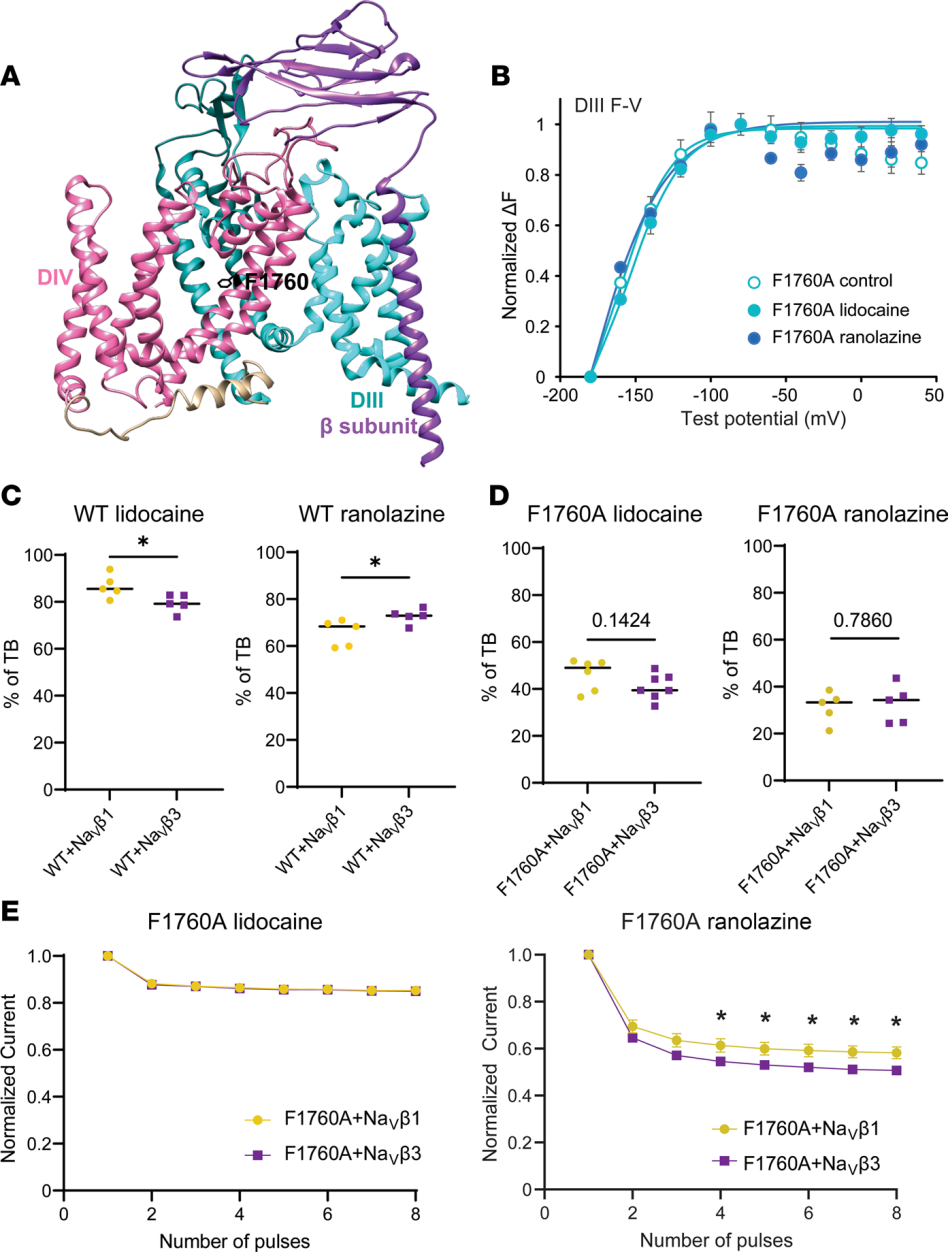
Altering the key local anesthetics’ binding site F1760 did not completely abolish Na_V_β1/β3 modulations of ranolazine block. (**A**) Cryo-electron microscopy structure of human Na_V_1.4(11) (Protein Data Bank 6AGF) showing the relative locations of the F1760 residue, DIII, DIV, and Na_V_β1. (**B**) Mutating the main local anesthetic binding residue F1760 to alanine (**A**) greatly reduced the hyperpolarizing shift in the DIII-VSD upon 10 mM lidocaine, as well as 4 mM ranolazine, observed in the WT channel (Figures 1 and 2). (**C**) Percentage of TB induced by 10 mM lidocaine and 4 mM ranolazine in the WT channel. The presence of Na_V_β3 reduced lidocaine TB but enhanced ranolazine TB compared with the α-Na_V_β1 complex. (**D**) Percentage of TB induced by 10 mM lidocaine and 4 mM ranolazine in the F1760A channel. In contrast to WT, Na_V_β1 and Na_V_β3 no longer exerted a significant effect on lidocaine and ranolazine TB. (**E**) UDB by lidocaine and ranolazine in F1760A channel coexpressed with Na_V_β1 or Na_V_β3. There was no change in lidocaine UDB comparing coexpression with Na_V_β1 and Na_V_β3. However, the presence of Na_V_β1 caused a reduced ranolazine UDB compared with Na_V_β3, a phenomenon that is similar to Na_V_β1’s effects on the WT channel. Each data set represents mean ± SEM values from 3–6 cells. Unpaired 2-tailed Student’s *t* test was used to test significance (**C**–**E**). *P < 0.05.

These results demonstrated that Na_V_β subunits differentially regulated lidocaine and ranolazine interactions with the DIII-VSD in heterologously expressed Na_V_1.5 channels. Specifically, Na_V_β1 enhanced the effect of lidocaine but decreased the effect of ranolazine on the DIII-VSD activation, whereas Na_V_β3 coexpression had the opposite effects on both drugs. The altered drug interactions with the DIII-VSD resulted in an enhanced block by lidocaine and reduced block by ranolazine when the Na_V_1.5 α subunit was coexpressed with Na_V_β1 compared with Na_V_β3 (Figure 3C).

To determine whether the differential modulation of lidocaine and ranolazine block by Na_V_β1 and Na_V_β3 is dependent on the main local anesthetic binding site F1760 (20, 31) (Figure 3A), we assessed drug blockade of the F1760A-mutant Na_V_1.5 channel in the presence of Na_V_β1 or Na_V_β3. As expected, the F1760A-mutant channels exhibited much reduced block by lidocaine and ranolazine compared with the WT channels (Figure 3, C and D). However, application of 10 mM lidocaine or 4 mM ranolazine still caused significant tonic block (TB) and use-dependent block (UDB) of the F1760A channels (Figure 3, D and E). TB reflects resting-state drug block, while UDB requires prior channel opening (32). In contrast to the WT channels, the hyperpolarizing shift in the DIII F-V upon lidocaine or ranolazine block was not observed with the F1760A-mutant channels (Figure 3B). The F1760A mutation also eliminated Na_V_β1 and Na_V_β3 modulation of TB by lidocaine and ranolazine as well as UDB by lidocaine (Figure 3, D and E). However, despite the absence of a major drug-binding site, coexpression of Na_V_β3 still caused stronger UDB by ranolazine compared with Na_V_β1 (Figure 3E). These results suggest that the effects of Na_V_β1/β3 on lidocaine and ranolazine block are affected by the F1760 anesthetic binding site but are not completely dependent on it.

### Loss of Scn1b expression in mouse cardiomyocytes does not affect Na_V_ channel gating.

**Figure 4:**
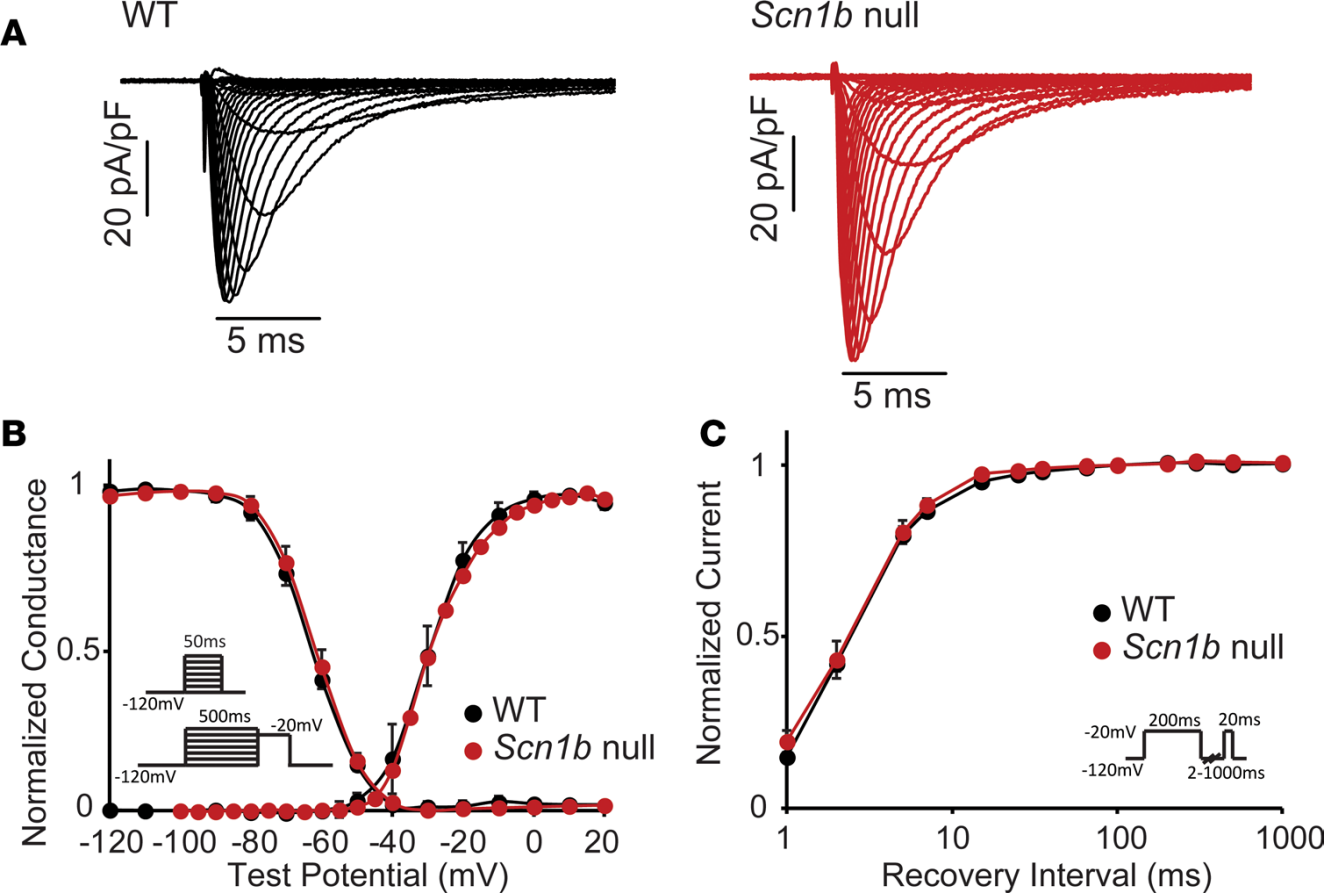
I_Na_ gating is similar in *Scn1b*-null and WT mouse LV myocytes. (**A**) Representative recordings of I_Na_ in WT and *Scn1b*-null mouse LV myocytes revealed similar kinetics of activation and inactivation. However, the average peak current density was slightly (~28%) higher in *Scn1b*-null compared with WT. (**B**) Loss of Na_V_β1 in *Scn1b*-null mouse LV myocytes did not affect the voltage dependences of I_Na_ activation or steady-state inactivation. (**C**) Loss of Na_V_β1 in *Scn1b*-null mouse LV myocytes also did not affect the time course of I_Na_ recovery from inactivation. Each data set represents mean ± SEM values from 6–9 cells.

To further investigate how noncovalent Na_V_β1/β1B subunits affect the cardiomyocyte response to class Ib antiarrhythmics, we utilized the cardiac-specific *Scn1b*-null mouse model (*Scn1b^fl/fl^/Myh6-cre*) described previously (28). First, we compared I_Na_ in left ventricular (LV) myocytes acutely dissociated from adult *Scn1b* cardiac-specific null and WT mice. Peak I_Na_ density was increased by 28% in *Scn1b-*null compared with WT LV myocytes (*Scn1b*-null: 81.3 ± 3.6 pA/pF, WT: 63.9 ± 5.2 pA/pF, *P* = 0.017). An increase in I_Na_ density in cardiac-specific *Scn1b*-null isolated from juvenile mice was previously reported (28). Consistent with the increase in current density, we also observed increased *Scn5a* transcript expression in the ventricles (and atria) of the *Scn1b*-null compared with WT mice (Supplemental Figure 1; Supplemental material available online with this article; https://doi.org/10.1172/jci.insight.143092DS1). Other than increasing peak current density, *Scn1b* deletion did not measurably alter other Na_V_ channel gating properties in ventricular cardiomyocytes (Figure 4A), including the voltage dependences of channel activation (Figure 4B), steady-state inactivation (Figure 4B), and/or the kinetics of channel recovery from inactivation (Figure 4C). Notably, deleting *Scn1b* did not measurably alter the expression of other Na_V_β subunits (Supplemental Figure 1). These results, although contrary to previously reported effects of Na_V_β1 on I_Na_ in heterologous expression systems, are consistent with results obtained in studies on global and cardiac-specific *Scn1b*-null mice (28, 33).

### Increased block of I_Na_ by ranolazine but reduced block by lidocaine in adult Scn1b-null mouse ventricular myocytes.

Even though Na_V_ channel gating was not measurably affected in cardiac-specific *Scn1b*-null myocytes, we went on to determine whether the loss of *Scn1b* affects the responses of native Na_V_ channels to class Ib antiarrhythmics. We examined the effects of lidocaine and ranolazine on TB and UDB of I_Na_ in LV myocytes isolated from WT and cardiac-specific *Scn1b*-null mice.

**Figure 5:**
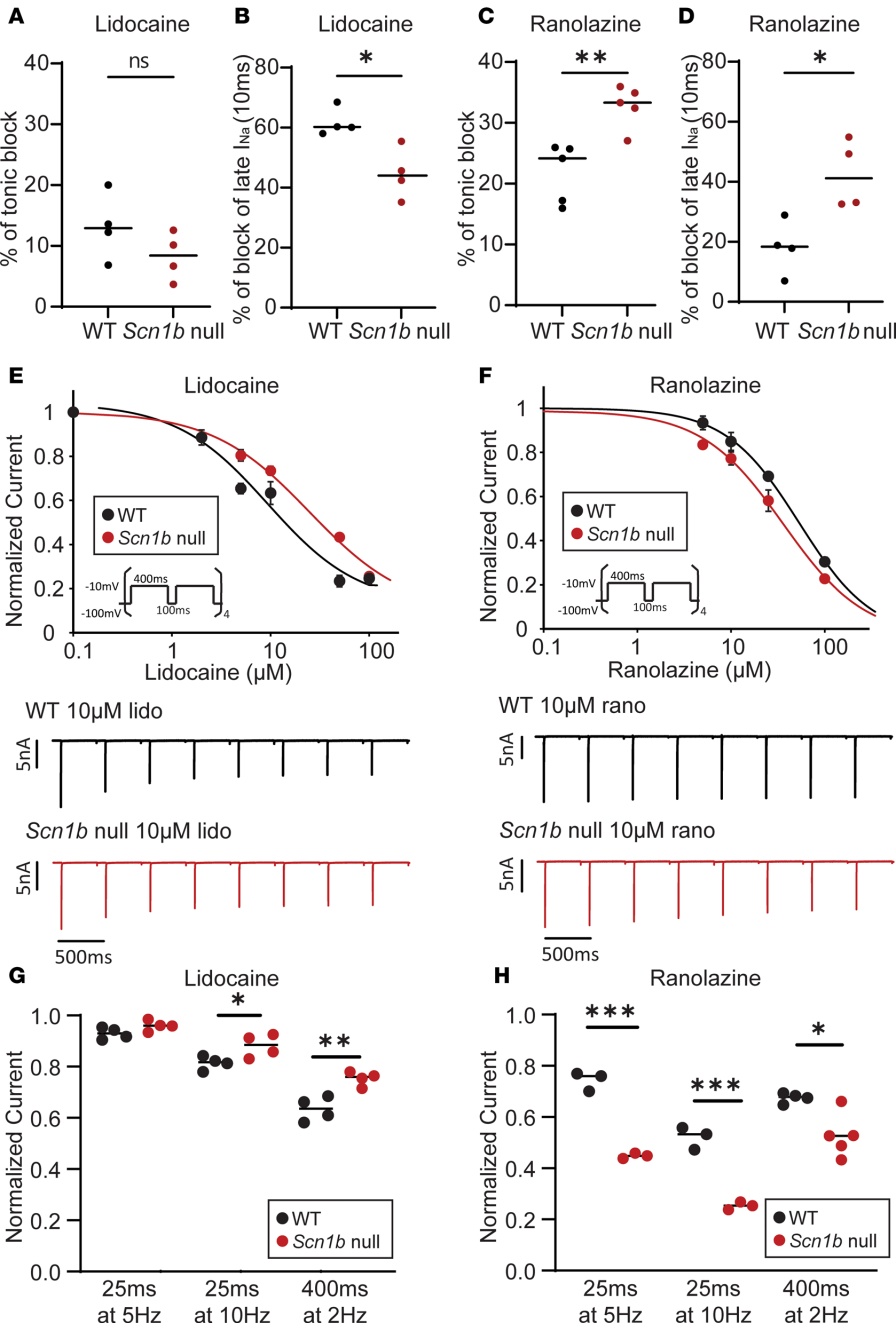
*Scn1b*-null LV myocytes show reduced lidocaine but enhanced ranolazine responses. (**A**) TB of I_Na_ by 100 μM lidocaine was slightly reduced in *Scn1b*-null compared with WT mouse LV myocytes. (**B**) Percentage of late I_Na_ block by 100 μM lidocaine was markedly lower in *Scn1b*-null compared with WT mouse LV myocytes. Late I_Na_ was measured 30 ms after the onset of the depolarizing voltage step. (**C**) TB of I_Na_ by 100 μM ranolazine was greater in *Scn1b*-null compared with WT mouse LV myocytes. (**D**) Percentage of late I_Na_ block by 100 μM ranolazine was greater in *Scn1b*-null compared with WT mouse LV myocytes. (**E**) Dose-response curve (top) and example traces (bottom) for UDB of I_Na_ by lidocaine. UDB was examined by measuring I_Na_ evoked in response to 8 repetitive (400 ms duration) depolarizations presented at 2 Hz, which determines the initial rate of UDB. The EC_50_ for UDB of I_Na_ by lidocaine was lower in WT compared with *Scn1b*-null suggesting that Na_V_β1 enhances the sensitivity to lidocaine. (**F**) Dose-response curve (top) and example traces (bottom) for UDB of I_Na_ by ranolazine. In contrast to lidocaine, the EC_50_ for UDB by ranolazine was higher in WT compared with *Scn1b*-null, suggesting Na_V_β1 reduces the effects of ranolazine. (**G**) Frequency-dependent UDB block of I_Na_ by 10 μM lidocaine in WT and *Scn1b*-null LV myocytes. UDB was assessed by measuring I_Na_ evoked by repetitive depolarizing pulses at 5 Hz (25 ms, 40 pulses), 10 Hz (25 ms, 40 pulses), and 2 Hz (400 ms, 8 pulses). Normalized currents indicate I_Na(last-pulse)_/I_Na(first-pulse)_. (**H**) Frequency-dependent UDB block of I_Na_ by 10 μM ranolazine in WT and *Scn1b*-null LV myocytes. Each data set represents mean ± SEM of data from 3–5 cells. Unpaired 2-tailed Student’s *t* test. *P < 0.05; **P < 0.01; ***P < 0.001.

The TB produced by 100 μM lidocaine was similar in WT and *Scn1b*-null LV myocytes (Figure 5A). In marked contrast, the block of late I_Na_ by lidocaine was significantly reduced in *Scn1b*-null LV myocytes (Figure 5B). There was also an approximately 3-fold reduction in lidocaine UDB in *Scn1b*-null compared with WT LV myocytes (WT: EC_50UDB_ = 9.3 μM, *Scn1b*-null EC_50UDB_ = 24.8 μM) (Figure 5E). Conversely, ranolazine increased TB, late I_Na_ block, and UDB in *Scn1b*-null compared with WT adult mouse LV myocytes (Figure 5, C, D, and F) (WT: EC_50UDB_ = 53.3 μM, *Scn1b*-null EC_50UDB_ = 36.0 μM). The differences in UDB by lidocaine between WT and *Scn1b*-null myocytes depended on the frequency and duration of the depolarizing pulses (Figure 5G). In response to 10 μM lidocaine, I_Na_ from *Scn1b*-null showed decreased UDB compared with WT myocytes at 10 Hz (25 ms duration) and 2 Hz (400 ms duration) but not 5 Hz (25 ms duration) (Figure 5G). In contrast, in response to 10 μM ranolazine, I_Na_ in WT myocytes showed increased UDB compared with Scn1b-null myocytes at all 3 frequencies (Figure 5H). Both lidocaine and ranolazine are known to cause a hyperpolarizing shift in the voltage dependence of steady-state inactivation of cardiac I_Na_ (24, 34), indicating that drug binding promotes channel inactivation at more hyperpolarized membrane potentials. Therefore, we also compared the voltage dependence of I_Na_ inactivation in WT and *Scn1b*-null LV myocytes before and after lidocaine or ranolazine application. These experiments revealed 100 μM lidocaine induced a hyperpolarizing shift in I_Na_ inactivation in WT LV myocytes and a smaller shift in *Scn1b*-null LV myocytes (Supplemental Figure 2A). Conversely, 100 μM ranolazine induced a comparable leftward shift in the voltage dependence of inactivation of I_Na_ in WT and *Scn1b*-null LV myocytes (Supplemental Figure 2B).

Overall, these cellular studies revealed that in adult mouse LV myocytes, the cardiac deletion of *Scn1b* resulted in reduced lidocaine UDB but increased ranolazine UDB. These results are consistent with our VCF data (Figures 1 and 2), suggesting that the presence of Na_V_β1 subunits enhanced lidocaine’s effects but reduced ranolazine’s effects on the Na_V_1.5 DIII-VSD. The reduced effects on the DIII-VSD are also consistent with the decreased UDB of I_Na_ observed in LV myocytes (Figure 5).

### Ranolazine and lidocaine induced distinct ECG phenotypes in WT and Scn1b-null mice.

**Figure 6:**
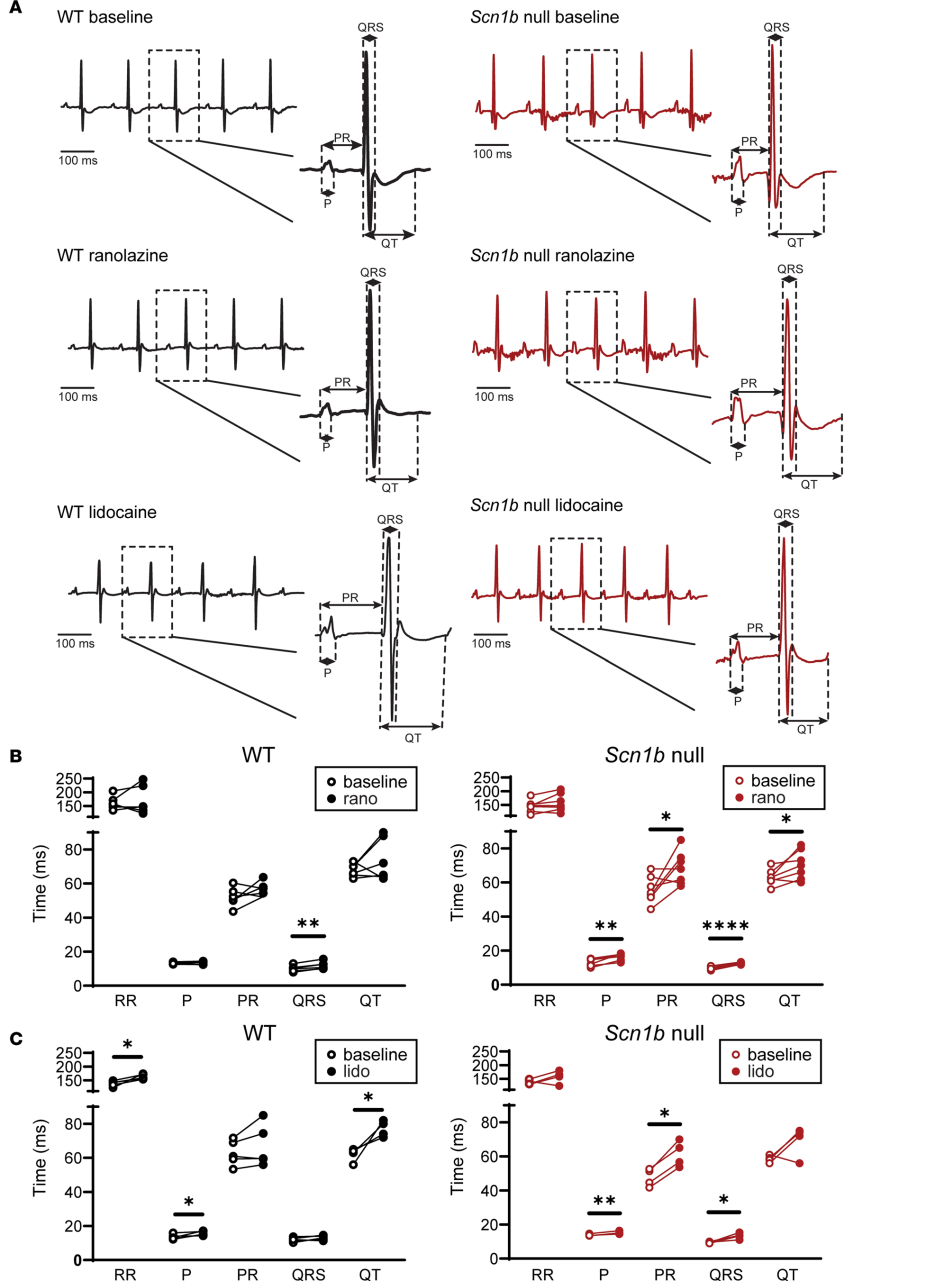
ECG recordings from WT and *Scn1b*-null mice before and after ranolazine or lidocaine injections. (**A**) Representative ECG recordings obtained from WT and *Scn1b*-null mice at baseline, postranolazine, and postlidocaine are presented. The postranolazine and postlidocaine data were recorded 10 minutes after the i.p. injections of ranolazine or lidocaine. P wave durations, PR, QRS, and QT intervals were measured as indicated in the insets. (**B**) Comparison of ECG parameters measured in WT (left panel) and *Scn1b*-null (right panel) mice at baseline and 10 minutes after i.p. injections of ranolazine injection. Ranolazine markedly prolonged the P wave duration and the PR interval in *Scn1b*-null but not in WT mice. (**C**) Comparison of ECG parameters measured in WT (left panel) and Scn1b-null (right panel) mice at baseline and 10 minutes after i.p. injections of lidocaine. Lidocaine markedly prolonged the RR interval, P wave duration, and QT interval in WT mice. In *Scn1b*-null mice, lidocaine also prolonged the P wave duration and resulted in marked prolongation of the PR and QRS intervals. Each data set represents data from 4–7 mice. The ECG parameters and statistical comparisons are shown in Supplemental Table 1.

To understand how Na_V_β1/β1B modulate antiarrhythmic responses at the whole-heart level, we measured surface ECGs in anesthetized WT and *Scn1b*-null mice before and after i.p. injection of lidocaine or ranolazine (Figure 6 and Supplemental Table 1). From the raw ECG data, we quantified several parameters that describe overall heart electrical functioning, including RR intervals, providing a measure of heart rates; P wave intervals, representing atrial conduction; PR intervals, characterizing atrial-ventricular conduction; QRS intervals, revealing ventricle conduction; and QT and ST intervals, corresponding to ventricular repolarization.

We found that 20 mg/kg ranolazine caused QRS prolongation in both WT and *Scn1b*-null mice (Figure 6, A and B), but that P wave and PR interval prolongation only occurred in the *Scn1b*-null mice (Figure 6B). The QT interval, but not the ST interval, was also prolonged by ranolazine in *Scn1b*-null mice (Figure 6B and Supplemental Figure 3). These results suggest that *Scn1b* deletion enhanced the inhibitory effect of ranolazine on cardiac conduction. Similar to the effects observed at the single myocyte level (Figures 4 and 5) that the loss of *Scn1b* enhanced TB and UDB of I_Na_ by ranolazine, loss of Na_V_β1/β1B in *Scn1b*-null mice promoted ranolazine block, manifesting as P wave, PR, and QRS interval prolongation.

We observed that 30 mg/kg lidocaine administration increased P wave duration in WT and *Scn1b*-null mice (Figure 6, A and C). In addition, lidocaine induced prolongation of RR, QT, and ST intervals in WT but not *Scn1b*-null mice (Figure 6C and Supplemental Figure 3). In contrast, lidocaine increased PR and QRS intervals in *Scn1b*-null mice (Figure 6C). Lidocaine injection, therefore, resulted in distinct functional effects in the 2 genotypes. We conducted control experiments in which we measured ECGs before and after injection of PBS solution. Comparison of baseline and post-PBS data showed that ECG parameters remained constant (Supplemental Figure 4).

### SCN1B is differentially expressed in human atria and ventricles.

**Figure 7:**
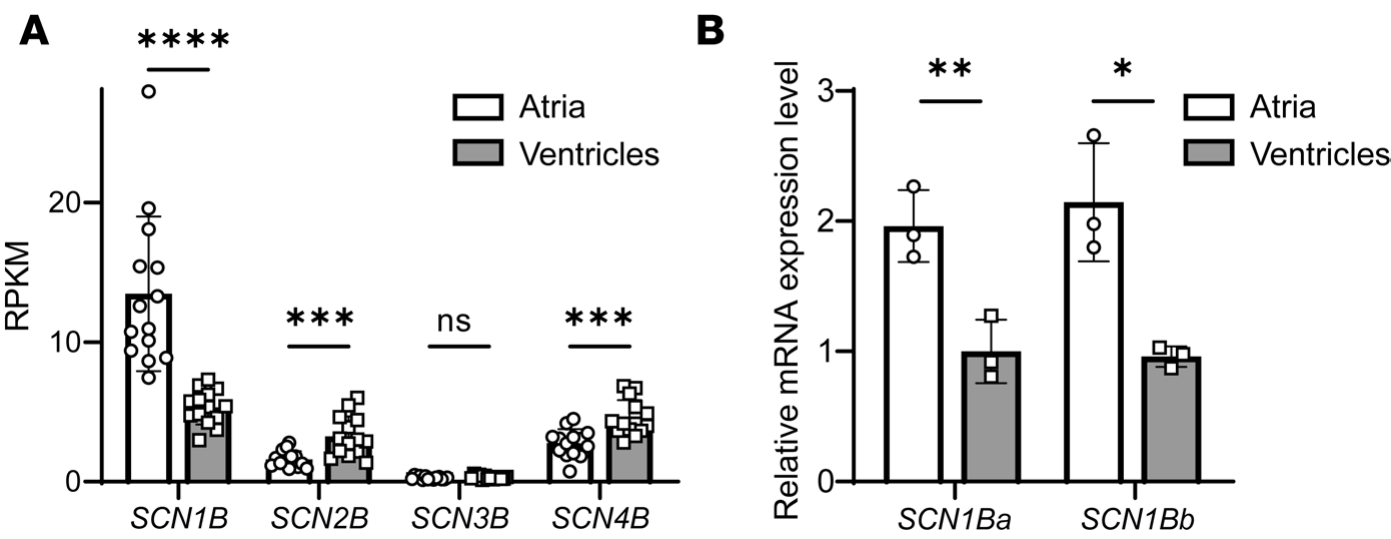
Regional differences in *SCN1B* expression in human atria and ventricles. (**A**) Extracted RNA-Seq data, expressed as reads per kilobase of exon per million mapped reads, from analyses of sequencing data obtained from matched (*n* = 8) human ventricular and atrial tissue samples (44). The *SCN1B* transcript was the most abundant of the Na_V_β subunits expressed in human atria and ventricles. In addition, *SCN1B* expression was approximately 3-fold higher in human atria compared with ventricles, whereas both *SCN2B* and *SCN4B* were approximately 2-fold higher in human ventricles than atria. (**B**) The differential expression of *SCN1B* in human atria and ventricles was confirmed by quantitative PCR (qPCR) analyses of the same paired human atrial and ventricular tissue samples analyzed by RNA-Seq. In addition, qPCR analyses using primers that distinguish the 2 *SCN1B* variants, *SCN1Ba* and *SCN1Bb*, revealed that the relative expression levels of both *SCN1Ba* and *SCN1Bb* transcripts were higher in the atria than the ventricles. Paired 2-tailed Student’s *t* test was used to test significance. *P < 0.05; **P < 0.01; ***P < 0.001; ****P < 0.0001.

The observation that loss of *Scn1b* alters the ability of class Ib antiarrhythmics to block Na_V_ channels in the mouse heart suggests that the differential expression of *SCN1B* might play an important role in regulating antiarrhythmic drug responses in humans. To begin to explore this hypothesis, we examined mRNA expression levels of the genes *SCN1B*, *SCN2B*, *SCN3B*, and *SCN4B* encoding Na_V_β subunits in human heart tissue in a recently published RNA-Seq library (35). These analyses revealed that in the human heart, *SCN1B* is the most abundant of the Na_V_β subunits at the transcript level and that *SCN1B* transcript expression is much higher in atria than in ventricles (Figure 7A). In contrast, the expression levels of the *SCN2B* and *SCN4B* transcripts are higher in the ventricles than in the atria (Figure 7A). To validate the RNA-Seq findings and to determine whether both *SCN1B* splice variants, *SCN1BA* (Na_V_β1) and *SCN1BB* (Na_V_β1B), are differentially expressed in human atria and ventricles, we performed quantitative RT-PCR analyses on the same tissue samples used in the RNA-Seq analyses. These experiments revealed that the relative expression levels of the 2 *SCN1B* splice variants were significantly higher in the atria compared with the ventricles (Figure 7B). Additional analyses revealed that, although expression of *SCN1BA* was approximately 100-fold higher than *SCN1BB*, the expression levels of the 2 (*SCN1BA* and *SCN1BB*) splice variants were similar in human right and left atria and in the LV and right ventricle (RV) (Supplemental Figure 5).

## Discussion

Although class Ib antiarrhythmics have considerable therapeutic potential, they are not broadly prescribed because of proarrhythmic risks in some patients and ineffectiveness in others (36, 37). Patient or disease variability in class Ib drug response suggests that there are external factors that modulate drug interactions with the channel (37). In this study, we investigated the role of Na_V_ channel accessory subunits Na_V_β1 and Na_V_β3 in regulating class Ib antiarrhythmic–mediated effects on Na_V_1.5 channels. We demonstrated that at a molecular level, Na_V_β1 or Na_V_β3 subunit coexpression differentially altered the effects of lidocaine and ranolazine on the Na_V_1.5 DIII-VSD. Na_V_β1 enhanced lidocaine but inhibited ranolazine modulation of the DIII-VSD. Conversely, Na_V_β3 eliminated lidocaine modulation but increased the effect of ranolazine on the DIII-VSD. Differential molecular interactions between Na_V_1.5 DIII-VSD and class Ib antiarrhythmic drugs caused by Na_V_β1 subunit expression in a heterologous system translated to distinct drug blockade of Na_V_ channels in WT versus *Scn1b* cardiac-specific null mouse cardiomyocytes. We further demonstrated differential effects of lidocaine and ranolazine on the ECG phenotypes of WT and *Scn1b*-null mice.

### Na_V_β1 and Na_V_β3 subunits alter Na_V_1.5 channel pharmacology via the DIII-VSD.

The DIII-VSD plays an important role in regulating Na_V_1.5 channel gating. It is involved in both activation and inactivation of Na_V_ channels (38, 39). Recent studies showed a correlation between DIII-VSD deactivation and the slow component of recovery from inactivation, which suggests that an activated form of the DIII-VSD stabilizes inactivation (39). Given that class Ib antiarrhythmics promote DIII-VSD activation, they may subsequently promote inactivation to induce greater levels of UDB. We recently demonstrated that DIII-VSD activation determines Na_V_1.5 channel blockade by mexiletine and affects the sensitivity of LQT3 variants to this drug (18), demonstrating a clear connection between the conformation of the DIII-VSD and class Ib drug potency.

We previously demonstrated that Na_V_β3 directly modulates the DIII-VSD, whereas Na_V_β1 does not (14). Recent channel structures suggest that Na_V_β1 associates with Na_V_1.7 and Na_V_1.4 through the DIII-VSD, an interaction that is not conserved in Na_V_1.5 (10, 12, 13). In light of these structural and functional data, it is plausible that Na_V_β3 interacts with Na_V_1.5 through similar sites as illustrated in the Na_V_1.4/ Na_V_1.7-β1 complex, while Na_V_β1 occupies a different site. Two distinct interaction mechanisms will result in the differential modulation of the DIII-VSD by Na_V_β1 and Na_V_β3. Here, we showed that coexpression of the Na_V_β1 or Na_V_β3 subunit differentially modulated the ability of class Ib antiarrhythmics to stabilize the DIII-VSD in the activated conformation, providing further evidence that in contrast to other Na_V_ channel α subunits, Na_V_β1 and Na_V_β3 have distinct interactions with Na_V_1.5. The effect of lidocaine on the DIII-VSD has been previously shown (14) to regulate UDB, a critical feature of class Ib drugs, which renders them most potent when myocytes are being excited repeatedly during an arrhythmic event. We observed that ranolazine and lidocaine similarly affected Na_V_1.5 α subunit–encoded channels expressed in the absence of the Na_V_β subunits. However, with the Na_V_β1 subunit present, the lidocaine effect on the DIII-VSD was enhanced, whereas the ranolazine effect was blunted. Conversely, Na_V_β3 enhanced ranolazine-induced DIII-VSD stabilization while inhibiting the effect of lidocaine. The differential regulation of the DIII-VSD resulted in distinct effects on the potencies of lidocaine and ranolazine depending on which Na_V_β was present. Thus, despite the similarity of the chemical structures (Figure 1), the therapeutic responses to lidocaine and ranolazine were differentially modified by the coexpression of the Na_V_β1 or the Na_V_β3 subunit.

### Na_V_β1/β1B modulates class Ib antiarrhythmic responses from molecular to the whole-heart level.

In *Xenopus* oocytes, with VCF and cut-open voltage clamp recordings, we demonstrated that Na_V_β1 coexpression enhanced lidocaine’s but inhibited ranolazine’s effect on the DIII-VSD, which resulted in an increased lidocaine but decreased ranolazine block. In addition, we observed increased UDB and late I_Na_ block by lidocaine but opposite effects with ranolazine in WT compared with *Scn1b*-null mouse LV myocytes. This cellular difference further led to distinct phenotypes of the in vivo ECG recordings in response to lidocaine and ranolazine injections. For example, in response to ranolazine injection, the P wave duration and PR interval were prolonged in the *Scn1b*-null mice but not in the WT mice. This difference in ECG parameters reflects the cellular phenotype of enhanced ranolazine block of I_Na_ in *Scn1b-*null compared with WT LV myocytes. However, not all the ECG changes can be explained by the differences observed in myocyte I_Na_ recordings of atrial and ventricular myocytes, and they may be caused by Na_V_β1-mediated effects on other regions of the myocardium, such as the sinoatrial and atrioventricular nodes (40). Alternatively, Na_V_β1 may affect drug interactions with other ion channels that regulate cardiac excitation, as discussed further below.

### Differential expression of Na_V_β1/β1B in human atria and ventricles and chamber-specific drug responses.

Ranolazine was proposed as a candidate for atrial specific therapy for AF (24, 41). Studies in the canine heart showed that atrial and ventricular cardiomyocytes have distinct responses to ranolazine (24). In the atria, ranolazine prolongs the action potential duration measured at 90% repolarization (APD_90_) and effective refractory period (24). In contrast, in the ventricle, ranolazine shortens the APD_90_. Here, we demonstrated higher *SCN1Ba* (Na_V_β1) and *SCN1Bb* (Na_V_β1B) subunit mRNA expression levels in human atria compared with ventricles. While we showed that Na_V_β1 coexpression attenuated ranolazine block of I_Na_ in both *Xenopus* oocytes and mouse LV myocytes, higher levels of Na_V_β1 in human atria may result in similar attenuation of the effects of ranolazine, thus contributing to decreased ranolazine blockade compared with ventricles. Notably, ranolazine is also a blocker of *KCNH2*–encoded (HERG-encoded) repolarizing I_Kr_ channels (22). If ranolazine blockade of I_Na_ is reduced in atria, modulation of I_Kr_ may dominate, resulting in prolongation of APD specifically in atria. Thus, the heterogeneous expression of Na_V_β1 may play a role in the chamber-specific ranolazine response.

### Na_V_β subunit modulation of class Ib drug effects may underlie disease-specific drug responses.

A search of NCBI’s Gene Expression Omnibus (GEO) profile database (42) revealed that *SCN1B* is upregulated in ischemic cardiomyopathies in human heart [GEO GDS651 and GDS1362 (43)] and mouse heart failure models [GEO GDS411, GDS427 (44), and GDS3660 (45)] (Supplemental Figure 6). These data suggest that the expression of Na_V_β subunits can be altered in disease remodeling of the cardiac tissue. Since late I_Na_ was found to be increased in the failing heart, class Ib drugs have become potential therapeutic approaches in targeting heart failure–related arrhythmias (46). As we have demonstrated that Na_V_β can differentially modulate class Ib effects, upregulation of Na_V_β1 in failing tissue may alter the patient response to Na_V_ channel–targeting antiarrhythmic therapies.

### Differential effects of Na_V_β subunits on lidocaine and ranolazine interactions reflect distinct molecular drug-pore interactions.

Previous work postulated that both lidocaine and ranolazine bind to common residues in the Na_V_1.5 channel pore (20, 31). However, we observed that lidocaine modulated both the DIII- and DIV-VSDs of Na_V_1.5, whereas ranolazine only affected the DIII-VSD when Na_V_1.5 was expressed without Na_V_β subunits, suggesting that these compounds may present in different orientations in the channel pore. Recent molecular dynamics simulations shed light on the detailed binding conformations of lidocaine and ranolazine within the Na_V_1.5 channel (47). Interestingly, these studies revealed that 2 lidocaine molecules can bind to the pore concurrently, one to the F1760 site and the other to the central pore (47). In contrast, ranolazine binds to the F1760 residue and possesses a more flexible linear structure, allowing it to interact with a larger area ranging from the fenestration to the selectivity filter (47). Moreover, ranolazine has a pK_a_ of 7.2 (48) and lidocaine has a pK_a_ of 7.9 (49). At physiological pH, therefore, a higher percentage of ranolazine molecules are expected to be uncharged compared with lidocaine. This difference will determine the relative percentages of the drug molecules entering in the hydrophobic pathway through the fenestration versus the hydrophilic pathway via the intracellular gate. The presence of Na_V_β1 or Na_V_β3 modulates DIII-VSD and DIV-VSD dynamics, which can allosterically affect the conformation of the pore and fenestrations. We demonstrated that Na_V_β1/β3’s modulation of ranolazine block was not entirely dependent on the main local anesthetic binding site, F1760, suggesting the changes in the DIII-VSD conformation due to Na_V_β modulation are essential for determining drug accessibility to the pore, independent of binding. In contrast, eliminating the F1760 binding site abolished the effects of Na_V_β1/β3 coexpression on lidocaine block, further suggesting that lidocaine acts through mechanisms distinct from those of ranolazine. Because lidocaine and ranolazine have different stoichiometries, orientations within the pore, and entrance pathways, it is plausible that changing channel VSD and pore conformations in the presence of Na_V_β subunits can result in opposite effects on DIII-VSD interactions with these drugs.

### Na_V_β subunit modulation of antiarrhythmic drug outcome beyond Na_V_ channels.

Aside from Na_V_ channels, Na_V_β subunits have been shown to modulate expression and gating of various potassium channels, including the voltage-gated K_V_ 4.3 and the inward-rectifying K_ir_ 2.1 channels, resulting in modifications of 2 essential cardiac currents, the fast transient outward K^+^ current (I_to,f_) and the inward rectifier current (I_K1_–) (50–52). Although unexplored to date, it also seems highly likely that the presence of Na_V_β subunits can affect K_V_ and K_ir_ channel pharmacology as well. Here, we showed that although single cardiac-specific *Scn1b*-null LV myocytes displayed attenuated inhibition of I_Na_ by lidocaine compared with WT cells, enhanced PR and QRS interval prolongation was observed in *Scn1b*-null animals with lidocaine, suggesting contributions from other cardiac ionic currents.

### Limitations.

To assess the drug effects on VSD dynamics, we conducted the VCF experiments in *Xenopus* oocytes. Because this is a heterologous expression system and the experiments were performed at 19°C, extrapolating the results to mammalian physiology can be difficult. However, we observed consistent drug-mediated modulatory effects in oocytes and in mouse LV myocytes, observations that support the hypothesis that the molecular mechanisms we identified with VCF are operative in mammalian systems.

We were only able to show that the expression of *SCN1B* was enriched in the atria compared with ventricles in the human heart at the transcript level. Although we attempted to examine protein expression levels directly, none of the available anti-Na_V_β1 antibodies detect Na_V_β1 proteins in mouse or human cardiac tissues. Since it is currently not possible to perform gene editing or to use siRNA-mediated knockdown strategies in native human myocytes, we were not able to explore the functional effects of Na_V_β1 on class Ib drugs in human myocytes directly.

### Conclusions.

In summary, we have demonstrated roles for noncovalently linked Na_V_β subunits in regulating antiarrhythmic drug effects from molecular interactions to whole-heart phenotypes. Our results elucidated the differential regulation of Na_V_1.5 channels by 2 class Ib agents, lidocaine and ranolazine, by Na_V_β1 and Na_V_β3 subunits. The unique expression profile of Na_V_β subunits in the human heart suggests chamber-dependent responses to these 2 compounds. Our findings provide crucial insights into strategies for improving the clinical outcomes of patients treated with class Ib agents for different forms of arrhythmias. Moreover, Na_V_β1 expression is upregulated in heart failure, and it remains unexplored whether Na_V_β subunit expression is affected in other heart pathologies. This knowledge will be highly valuable in establishing disease-specific approaches to personalize arrhythmia treatment with lidocaine and ranolazine because known changes in β subunit expression will have predictable effects on therapeutic outcomes.

## Methods

### Experimental animals.

Adult (8–15 weeks old) male and female WT and cardiac-specific *Scn1b-*null C57BL/6J mice were used in the experiments here. Cardiac-specific *Scn1b-*null mice were generated by crossing *Scn1b^fl^* mice (27) with *B6.FVBTg*(*Myh6-cre*)*2182Mds/J* mice (The Jackson Laboratory), which express Cre recombinase driven by the α-myosin heavy chain promoter. Mice were genotyped by PCR analyses of genomic (tail) DNA using primers targeting sequences external to the *loxP* sites, as well as primers targeting Cre recombinase, as previously described (27). Because of the breeding strategy required to generate cardiac-specific *Scn1b-*null C57BL/6J mice, WT littermates were not generated. The WT mice used in the experiments presented here, therefore, were not littermate controls; rather they were WT C57BL/6J mice from our colony. Additional control experiments, however, were conducted on *Scn1b^fl^* and *B6.FVBTg*(*Myh6-cre*)*2182Mds/J* mice, which were determined to be indistinguishable electrophysiologically from WT C57BL/6J animals. *Xenopus* oocyte harvests were performed as described previously (53).

### Cut-open VCF.

VCF experiments were conducted using 4 previously developed Na_V_1.5 channel constructs (DI: V215C, DII: S805C, DIII: M1296C, DIV: S1618C) (53). Capped mRNAs were synthesized with the mMESSAGE mMACHINE T7 transcription kit (Life Technologies, Thermo Fisher Scientific) from the linearized pMAX vectors. VCF construct mRNA was injected alone or coinjected with *SCN1B* or *SCN3B* mRNA into *Xenopus* oocytes as previously described (14). VCF experiments were performed 4–6 days after injection. The recording setup and labeling protocol used were described previously (14, 53–55). Lidocaine hydrochloride and ranolazine dihydrochloride were dissolved in extracellular recording solution and then further diluted to 10 mmol/L and 4 mmol/L, respectively. Both drugs were manually perfused into the extracellular solution chamber in the cut-open voltage clamp setup. Fluorescence signals and currents were analyzed as previously described (18). V_1/2_ values reported were quantified from the Boltzmann function fit, y = 1/(1 + exp [V – V_1/2_]/k). Because the DIII F-V curve, especially under drug treatment conditions, did not saturate at the lowest voltage recorded (–160 mV), the fit was performed by fixing 0 at –200 mV. Because of the lack of saturation at the most negative potentials measured, the estimated V_1/2_ for DIII F-V is likely to be higher than the actual V_1/2_ value.

### ECG recordings.

Surface ECG recordings were obtained as previously described from mice anesthetized by i.p. injection of avertin (0.25 mg/kg; MilliporeSigma) (56). Baseline ECGs were recorded, and animals were weighed. For injections, drugs were dissolved in 250 μL PBS. Lidocaine or ranolazine was then injected at a dosage of 30 mg/kg or 20 mg/kg, respectively. Different animals were used for lidocaine or ranolazine injections. Between recordings, mice were kept on a heating pad maintained at 37°C ± 0.5°C. Postinjection ECGs were recorded at 5 minutes, 10 minutes, 15 minutes, 20 minutes, and 30 minutes. Peak responses were observed at 10 minutes, which was subsequently selected as the time point for ECG analysis.

RR, PR, and QT intervals, as well as P and QRS durations, were measured and compiled using Clampfit 10.3 (Molecular Devices) and GraphPad Prism. Note that QT intervals shown in figures were not corrected because several recent studies have shown that QT intervals in anesthetized mice do not vary with heart rate (57, 58). Similar differences were revealed, however, when corrected QT intervals were compared (Supplemental Table 1).

### Isolation of adult mouse cardiomyocytes.

Myocytes were isolated from adult (8 to 12 weeks old) WT or *Scn1b-*null mice as previously described (59). Briefly, hearts were isolated and perfused retrogradely through the aorta with a Ca^2+^-free Earle’s balanced salt solution containing 0.8 mg/mL type II collagenase (Worthington). After perfusion, the LV free wall was dissected and minced. The tissue pieces were then triturated to provide individual LV myocytes. Dispersed cells were then filtered and resuspended in Medium199 (Gibco, Thermo Fisher Scientific), plated on laminin-coated (MilliporeSigma) glass coverslips, and maintained in a 95% air/5% CO_2_ incubator at 37°C.

Whole-cell Na_V_ current (I_Na_) recordings were obtained from isolated LV myocytes at room temperature (22°C–24°C) within 5–6 hours of isolation using a Dagan 3900A amplifier interfaced to a Digidata 1332A A/D converter (Axon) using pClamp 10.2 (Axon). Recording pipettes contained the following (in mmol/L): 120 glutamic acid, 120 CsOH, 10 HEPES, 0.33 MgCl_2_, 20 tetraethylammonium chloride (TEACl), 4 Mg-ATP, 5 glucose, and 5 EGTA (pH adjusted to 7.3 with CsOH); pipette resistances were 1.5–3.0 MΩ. The bath solution contained the following (in mM): 20 mM NaCl, 110 mM TEACl, 4 KCl, 2 MgCl_2_, 1 CaCl_2_, 10 HEPES, and 10 glucose (pH 7.4; 300 mOsm).

Electrophysiological data were acquired at 10–20 KHz, and signals were low-pass-filtered at 5 kHz before digitization and storage. After the formation of a giga-seal (>1 GΩ) and establishment of the whole-cell configuration, brief (10 ms) ± 10 mV voltage steps from a holding potential (HP) of –70 mV were presented to allow measurements of whole-cell membrane capacitances (C_m_), input resistances (R_in_), and series resistances (R_s_). In each cell, C_m_ and R_s_ were compensated electronically by approximately 85%; voltage errors resulting from uncompensated R_s_ were less than 2 mV and were not corrected. Leak currents were always less than 50 pA and were not corrected. Whole-cell I_Na_ were evoked in response to 40 ms voltage steps to potentials between –60 and +40 mV from an HP of –100 mV in 10 mV increments at 15-second intervals.

Electrophysiological data were compiled and analyzed using Clampfit 10.3 (Molecular Devices) and GraphPad Prism.

### Quantitative reverse-transcription PCR.

Total RNA (2 μg) isolated from individual matched (*n* = 6) human right atria, left atria, RV, and LV tissue samples was reverse-transcribed into cDNA with a high-capacity cDNA kit. Transcript analysis was conducted with SYBR green using a 7900HT Fast Real-Time PCR system (all from Applied Biosystems, Thermo Fisher Scientific). Data were analyzed using the Ct relative quantification method using the *GAPDH* and hypoxanthine guanine phosphoribosyl transferase I (*HPRT*) genes as endogenous controls.

### Statistics.

Results are presented as mean ± SEM. The number of animals and the number of cells used in each experiment are provided in the figure legends. Comparisons of differences between WT and *Scn1b*-null cells/animals under control conditions and before and after drug treatments were performed using a paired 2-tailed Student’s *t* test (Microsoft Excel). In comparisons of more than 2 groups, 1-way ANOVA was used followed by multiple comparisons. The *P* values shown were corrected for multiple hypothesis testing using Dunnett’s correction method. *P* values less than 0.05 were considered significant.

### Study approval.

All animals were handled in accordance with the NIH Guide for the Care and Use of Laboratory Animals, and all experimental protocols were approved by the Washington University IACUC.

## Author contributions

WZ contributed to designing studies, conducting experiments, acquiring data, analyzing data, and writing the manuscript. WW, PA, and RLM contributed to conducting experiments and acquiring data. LLI contributed to providing experimental animals and editing the manuscript. JMN and JRS contributed to designing studies and editing the manuscript.

## Supplementary Material

Supplemental data

## Version 1 06/22/2021

In-Press Preview

## Version 2 08/09/2021

Electronic publication
