# Supplementary material for: Modulation of the effects of class Ib antiarrhythmics on cardiac NaV1.5-encoded channels by accessory NaVβ subunits
Source: JCI Insight. 2021 Aug 9;6(15):e143092. doi: 10.1172/jci.insight.143092 (PMC8410097; doi:10.1172/jci.insight.143092)
Supplement: Supplemental data [file jciinsight-6-143092-s029.pdf]

Supplement Figure 1: qRT-PCR analyses of the transcripts encoding all Navβ subunits and the major Nav α subunits in the atria and left ventricles (LV) of WT and *Scn1b* null mice.

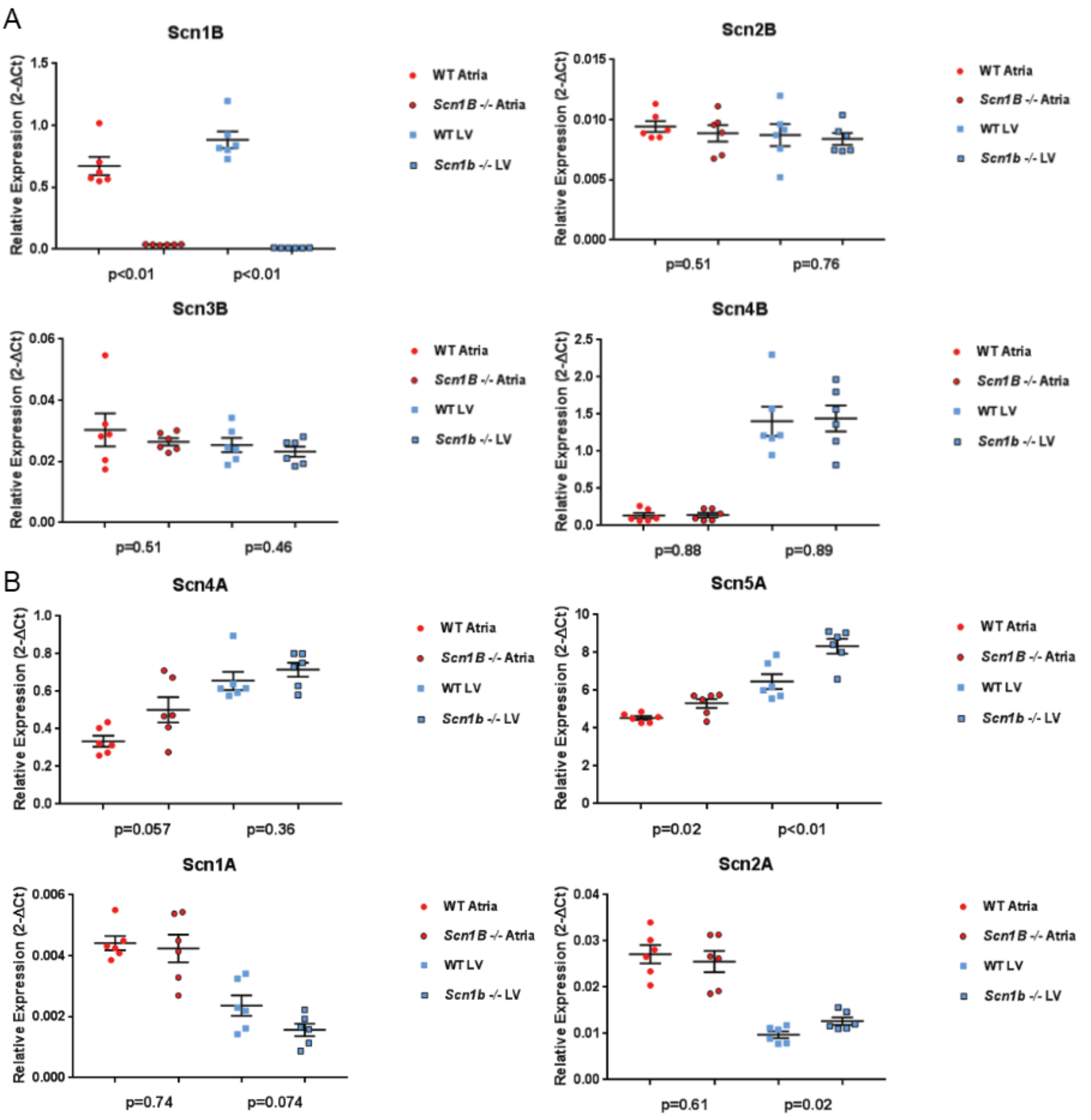

A. The *Scn1B* transcript is undetectable in the atria and LV of *Scn1b* null mice. The expression of the *Scn2B*, *Scn3B*, and *Scn4B* transcripts in *Scn1b* null and WT atria and LV are indistinguishable from WT atria and LV. B. Expression of the *Scn5A* transcript, however, is

higher in the atria and LV of *Scn1b* null, compared to WT, mice. *Scn2A* transcript expression is also increased in *Scn1b* null LV, compared with WT LV.

Supplement Figure 2: Effects of lidocaine and ranolazine on the voltage dependence of steady-state inactivation of I_{Na} in WT and *Scn1b* null mouse LV myocytes.

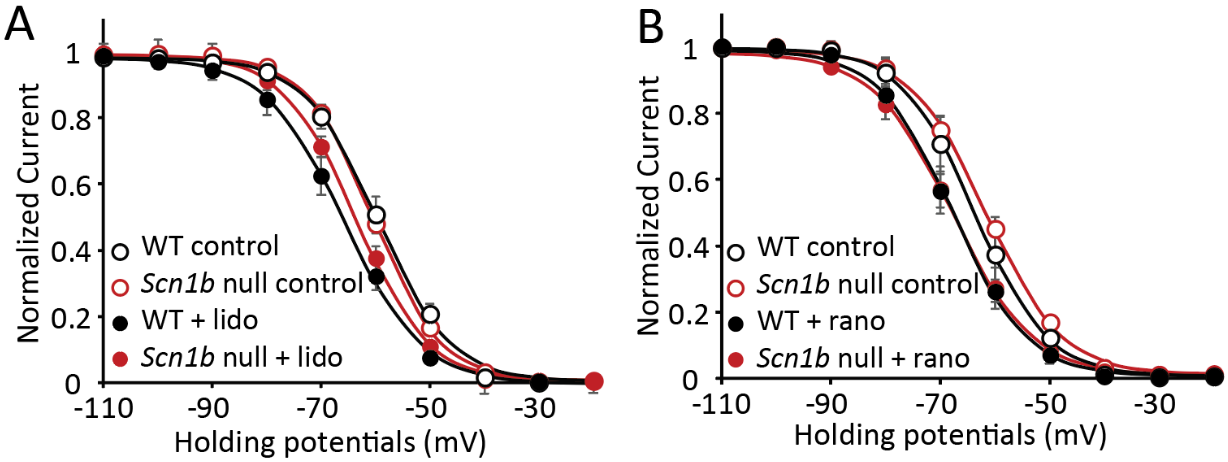

A. 100µM Lidocaine induced a small hyperpolarizing shift in the voltage dependence of I_{Na} inactivation in WT and Scn1b null LV myocytes, although the magnitude of the shift is smaller in *Scn1b* null myocytes. B. 100µM ranolazine resulted in similar small shifts in the voltage dependences of I_{Na} inactivation in WT and *Scn1b* null LV myocytes.

Supplement Figure 3: Quantified ST interval from ECG recordings obtained from WT and *Scn1b* null mice before and after IP injections of lidocaine or ranolazine.

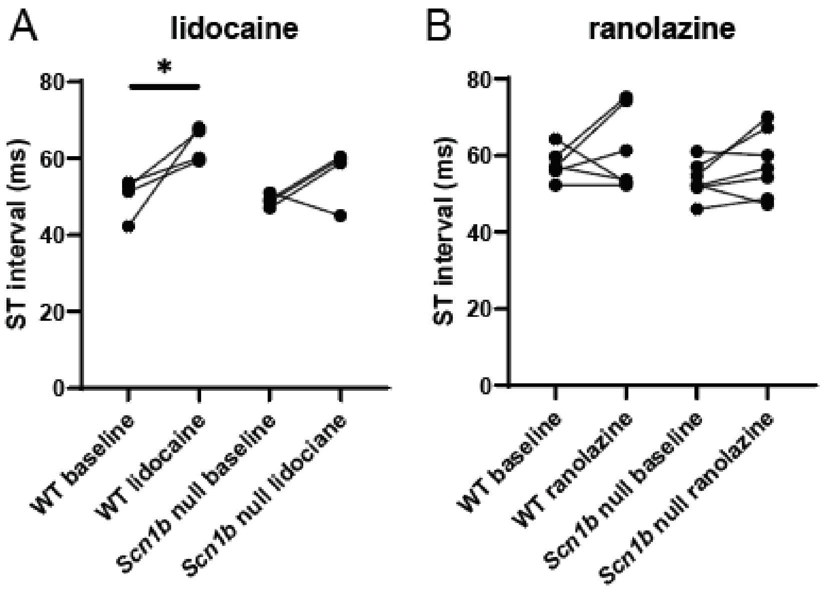

A. The ST interval is increased in WT, but not *Scn1b* null, mice in response to lidocaine. B. ST intervals are not measurably changed in either WT or Scn1b null mice after ranolazine injection.

Supplement Figure 4: ECG recordings obtained from WT and *Scn1b* null mice before and after IP injections of control (PBS) buffer.

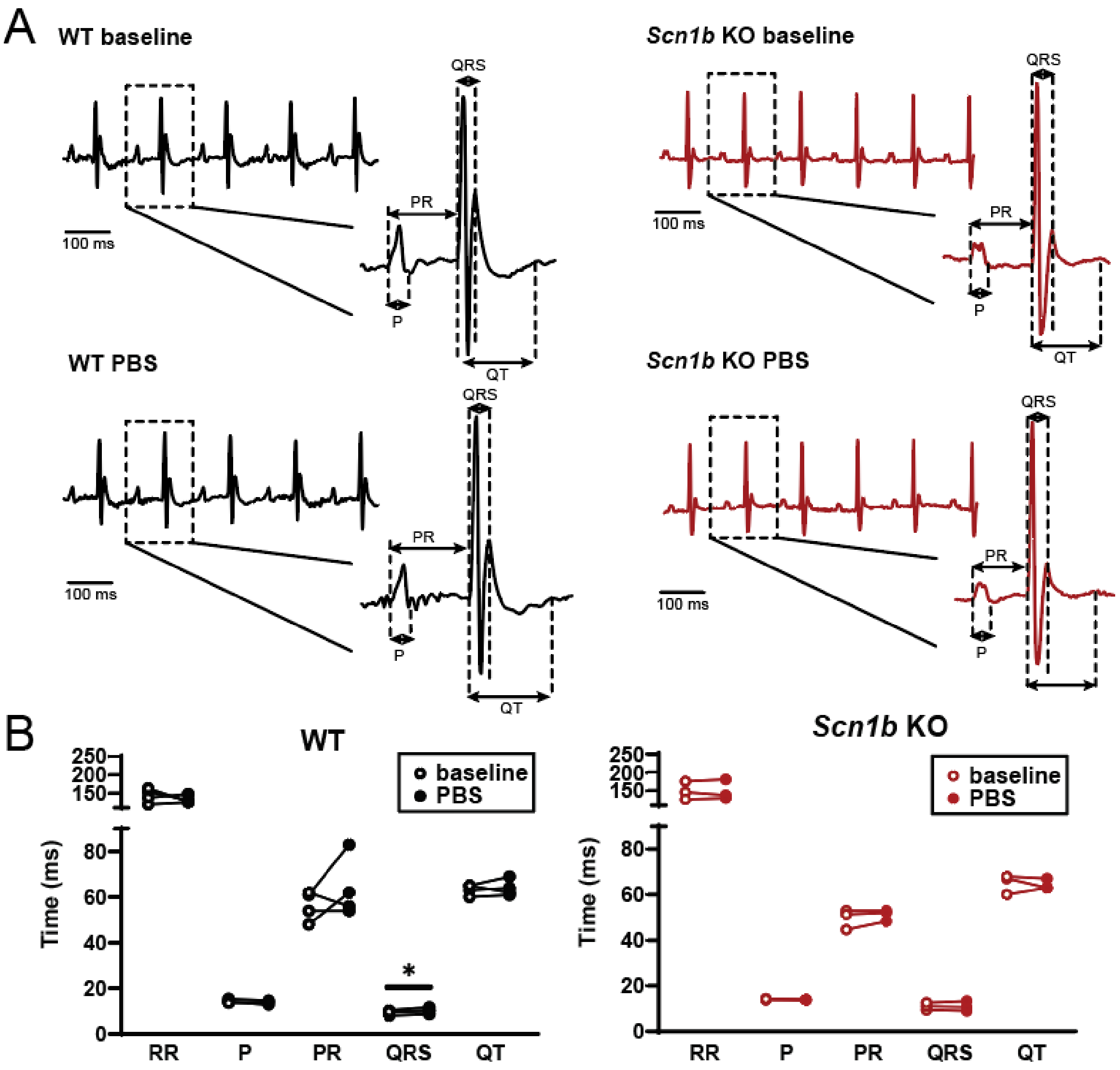

A. Representative ECG recordings from WT (black traces) and *Scn1b* null (red traces) mice at baseline and 10 minutes after IP injections of PBS. B. Quantification of ECG parameters, including RR intervals, P wave durations, PR, QRS, and QT intervals revealed that injection of PBS does not measurably affect ECG parameters.

Supplement Figure 5: qRT-PCR analyses of expression of the two *SCN1B* splice variants, *SCN1Ba and SCN1Bb*, in the four chambers of the human heart.

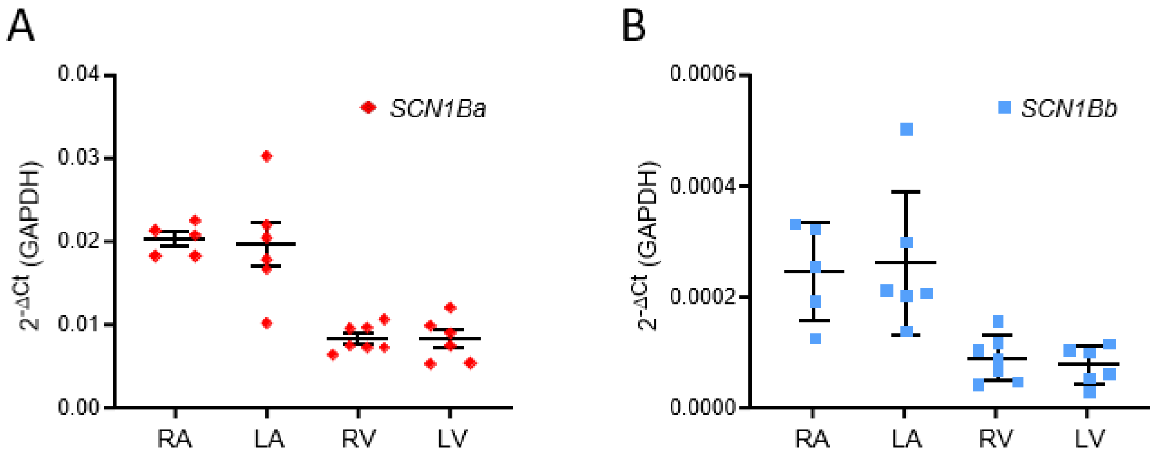

Note that the *SCN1Ba* transcript (A), which encodes the $Na_v\beta1$ protein, is expressed at ~ 100-fold higher levels than the *SCN1Bb* (B) transcript, which encodes the secreted $Na_v\beta1b$ protein. In addition, the expression levels of both the *SCN1Ba* (A) and the *SCN1Bb* (B) transcripts are higher in the right and left atria (RA and LA), compared to the right and left ventricles (RV and LV).

Supplement Figure 6: Expression profile of human *SCN1B* and mouse *Scn1b* in normal and failing ventricular tissues.

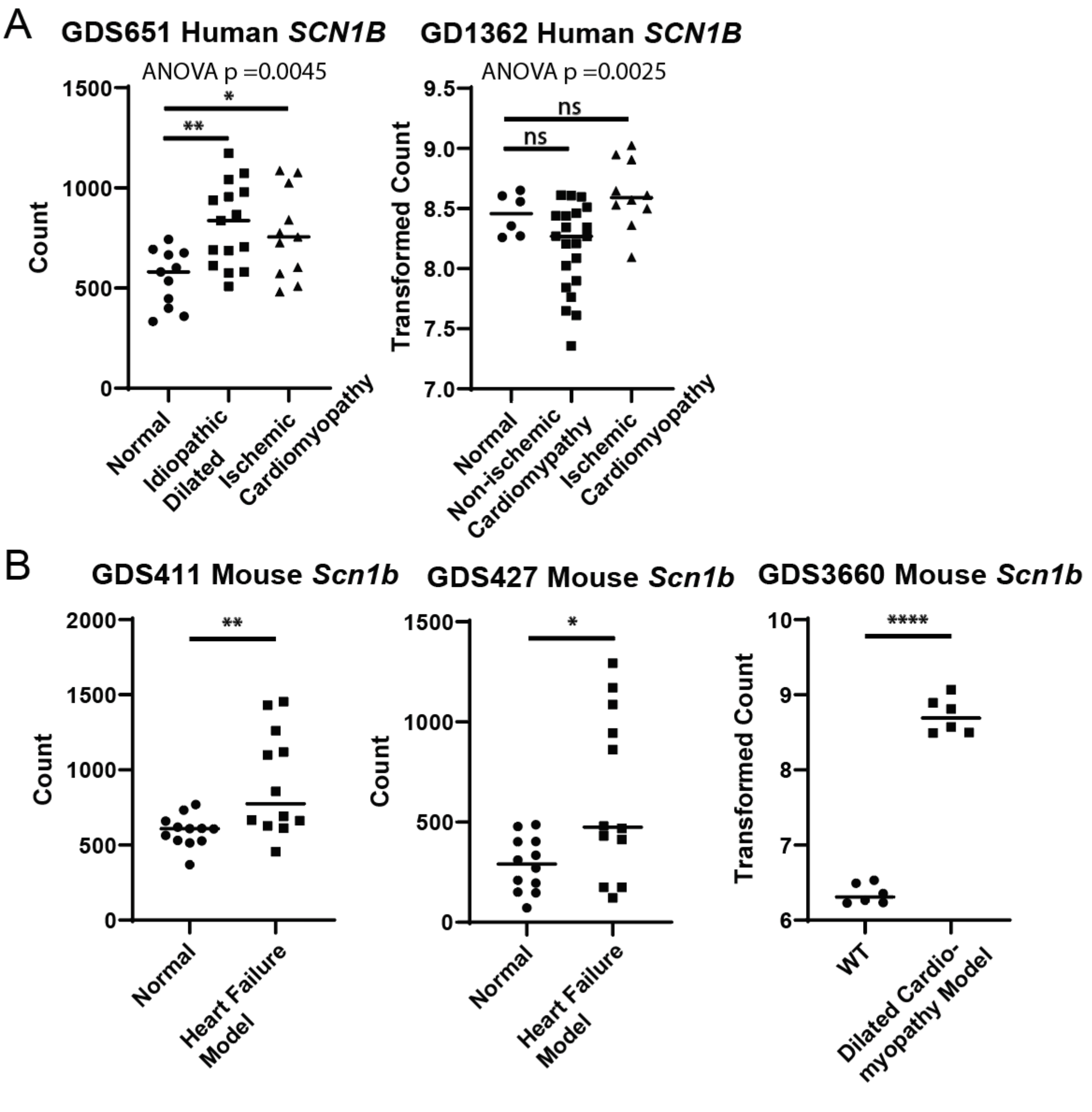

Microarray data showing *SCN1B* and *Scn1b* expression were extracted from the GEO Profile database (GEO accession GDS651, GDS1362, GDS411, GDS427, and GDS3660). All studies investigating dilated cardiomyopathy from the GEO database are shown here for comparison, with a filter of overall sample size greater than 20 and samples collected from the ventricles. A. Two studies assessing gene expression change in non-ischemic and ischemic cardiomyopathy in

human heart. Both studies indicated an increase in *SCN1B* expression in ischemic cardiomyopathy. B. *Scn1b* is upregulated in murine heart failure models compared to control. Genetic heart failure models were generated by deleting the muscle LIM protein gene (MLP-/-) or overexpressing the calsequestrin protein (GDS411 and GDS427). The dilated cardiomyopathy models used in GDS3660 were transgenic mice expressing a dominant negative CREB transcription factor.

Supplement Table 1: ECG parameters for WT and *Scn1b* null mice before and after ranolazine or lidocaine IP injection.

Mouse genotypes	**WT Total n = 13**					
ECG parameters (msec)	**Baseline**	**Ranolazine**	**p-value**	**Baseline**	**Lidocaine**	**p-value**
RR interval	161.3 ± 10.1	170.2 ± 21.2	0.601	139.0 ± 4.9	165.3 ± 3.8	0.116
P wave duration	13.1 ± 0.2	13.5 ± 0.4	0.233	13.5 ± 0.7	15.6 ± 0.5	0.009
PR interval	52.1 ± 2.3	56.2 ± 1.8	0.199	62.8 ± 3.3	66.8 ± 5.2	0.041
QRS duration	10.1 ± 0.7	12.0 ± 0.8	0.001	12.0 ± 0.6	13.0 ± 0.6	<0.0001
QT interval	67.8 ± 1.5	73.7 ± 5.0	0.269	62.0 ± 1.8	77 ± 2.1	0.035
QTc interval *	53.7 ± 1.4	56.9 ± 0.8	0.087	52.6 ± 0.8	59.9 ± 1.0	0.038

Mouse genotypes	***Scn1b* null Total n = 12**					
ECG parameters (msec)	**Baseline**	**Ranolazine**	**p-value**	**Baseline**	**Lidocaine**	**p-value**
RR interval	145.1 ± 8.4	158.1 ± 12.1	0.018	137.8 ± 4.5	157.0 ± 11.2	0.212
P wave duration	13.0 ± 0.8	16.2 ± 0.8	0.043	13.8 ± 0.3	15.2 ± 0.4	0.008
PR interval	55.9 ± 2.9	68.5 ± 3.6	0.200	47.6 ± 2.6	61.3 ± 3.7	0.004
QRS duration	9.6 ± 0.3	12.7 ± 0.2	0.177	9.5 ± 0.2	13.3 ± 0.9	0.029
QT interval	63.1 ± 1.7	70.4 ± 3.2	0.038	58.5 ± 1.0	69.3 ± 4.5	0.134
QTc interval *	52.6 ± 1.2	56.2 ± 0.9	0.016	50.3 ± 0.6	55.2 ± 1.6	0.092

* Corrected QT (QTc) intervals shown were calculated based on Bazett's formula adapted for mice $QTc = QT/(RR/f)^{1/2}$, f=100ms.
